# Supplementary material for: From sequence to enzyme mechanism using multi-label machine learning
Source: BMC Bioinformatics. 2014 May 19;15:150. doi: 10.1186/1471-2105-15-150 (PMC4229970; doi:10.1186/1471-2105-15-150)
Supplement: Additional file 2 — Java code of ml2db. Additional file ml2db_code.tar.gz contains the Java source code to run the multi-label machine learning experiments and save the results to database. The code’s Javadoc is included. [file 1471-2105-15-150-S2.zip › additional file 2/ml2db/ml2db/doc/mulan/transformations/multiclass/SelectRandom.html]

SelectRandom


JavaScript is disabled on your browser.


- Overview
- Package
- Class
- Use
- Tree
- Deprecated
- Index
- Help

- Prev Class
- Next Class

- Frames
- No Frames

- All Classes

- Summary:
- Nested |
- Field |
- Constr |
- Method

- Detail:
- Field |
- Constr |
- Method


mulan.transformations.multiclass

## Class SelectRandom

- java.lang.Object
- - mulan.transformations.multiclass.MultiClassTransformationBase
  - - mulan.transformations.multiclass.SelectRandom

- All Implemented Interfaces:
  :   java.io.Serializable, MultiClassTransformation

  ---

    

  ```
  public class SelectRandom
  extends MultiClassTransformationBase
  ```

  Class that implement the Select-Random transformation method

  Version:
  :   2012.02.02

  Author:
  :   Stavros Bakirtzoglou

  See Also:
  :   Serialized Form

- - ### Constructor Summary

    Constructors

    Constructor and Description

    `SelectRandom()`
  - ### Method Summary

    - ### Methods inherited from class mulan.transformations.multiclass.MultiClassTransformationBase

      `transformInstances`
    - ### Methods inherited from class java.lang.Object

      `equals, getClass, hashCode, notify, notifyAll, toString, wait, wait, wait`

- - ### Constructor Detail


    - #### SelectRandom

      ```
      public SelectRandom()
      ```


- Overview
- Package
- Class
- Use
- Tree
- Deprecated
- Index
- Help

- Prev Class
- Next Class

- Frames
- No Frames

- All Classes

- Summary:
- Nested |
- Field |
- Constr |
- Method

- Detail:
- Field |
- Constr |
- Method
